# Quinine Inhibits Infection of Human Cell Lines with SARS-CoV-2

**DOI:** 10.3390/v13040647

**Published:** 2021-04-09

**Authors:** Maximilian Große, Natalia Ruetalo, Mirjam Layer, Dan Hu, Ramona Businger, Sascha Rheber, Christian Setz, Pia Rauch, Janina Auth, Maria Fröba, Ekkehard Brysch, Michael Schindler, Ulrich Schubert

**Affiliations:** 1 Institute of Virology, Friedrich-Alexander University Erlangen-Nürnberg (FAU), 91054 Erlangen, Germany; Maximilian.Grosse@uk-erlangen.de (M.G.); Christian.Setz@uk-erlangen.de (C.S.); Pia.Rauch@uk-erlangen.de (P.R.); Janina.Auth@fau.de (J.A.); Maria.Carolin.Froeba@fau.de (M.F.); 2 Institute for Medical Virology and Epidemiology of Viral Diseases, University Hospital Tübingen, 72076 Tübingen, Germany; natalia.ruetalo-buschinger@med.uni-tuebingen.de (N.R.); mirjam.gneiting@med.uni-tuebingen.de (M.L.); dan.hu@med.uni-tuebingen.de (D.H.); ramona_businger@web.de (R.B.); 3 ImmunoLogik GmbH, 13507 Berlin, Germany; Sascha.Rheber@protonmail.com (S.R.); e.brysch@athenion.com (E.B.)

**Keywords:** SARS-CoV-2, quinine, COVID-19, antiviral

## Abstract

While vaccination campaigns are ongoing worldwide, there is still a tremendous medical need for efficient antivirals against SARS-CoV-2 infection. Among several drug candidates, chloroquine (CQN) and hydroxychloroquine (H-CQN) were tested intensively, and any contentious therapeutic effect of both has been discussed controversially in the light of severe side effects and missing efficacy. Originally, H-CQN descended from the natural substance quinine, a medicinal product used since the Middle Ages, which actually is regulatory approved for various indications. We hypothesized that quinine also exerts anti-SARS-CoV-2 activity. In Vero cells, quinine inhibited SARS-CoV-2 infection more effectively than CQN, and H-CQN and was less toxic. In human Caco-2 colon epithelial cells as well as the lung cell line A549 stably expressing ACE2 and TMPRSS2, quinine also showed antiviral activity. In consistence with Vero cells, quinine was less toxic in A549 as compared to CQN and H-CQN. Finally, we confirmed our findings in Calu-3 lung cells, expressing ACE2 and TMPRSS2 endogenously. In Calu-3, infections with high titers of SARS-CoV-2 were completely blocked by quinine, CQN, and H-CQN in concentrations above 50 µM. The estimated IC_50_s were ~25 µM in Calu-3, while overall, the inhibitors exhibit IC_50_ values between ~3.7 to ~50 µM, dependent on the cell line and multiplicity of infection (MOI). Conclusively, our data indicate that quinine could have the potential of a treatment option for SARS-CoV-2, as the toxicological and pharmacological profile seems more favorable when compared to its progeny drugs H-CQN or CQN.

## 1. Introduction

In the last two decades, three coronaviruses, SARS-CoV, MERS-CoV, and very recently, SARS-CoV-2, emerged from the animal kingdom, causing in each case life-threatening diseases in humans, particularly as non-immunocompetent populations were encountered. Infection with SARS-CoV-2 can cause the multi-organ disease COVID-19 (Coronavirus disease 2019), resulting, among other pathologies, in the acute respiratory distress syndrome (ARDS), a clinical condition of acute lung injury with severe hypoxemia [1]. Of all SARS-CoV-2-infected people, 80% remain asymptomatic or develop only mild symptoms, while others require hospitalization [2].

COVID-19 was declared as a worldwide public health emergency crisis in January with a pandemic dimension in March of 2020. As of 11.03.2021, over 118 million COVID-19 cases have been confirmed that have caused over 2.6 million deaths worldwide, according to Johns Hopkins University reports [3]. While vaccination campaigns are ongoing, the only antiviral therapeutic option approved in the U.S. for late-stage hospitalized patients remains remdesivir, a nucleotide analog originally developed to cure Ebola infection [4]. However, recent trials on remdesivir showed no real benefit [5]. In addition, there is evidence that plasma of convalescent patients [6] and application of certain monoclonal antibodies [7] can prevent outbreaks or support recovery from COVID-19. Furthermore, some repurposed drugs were in clinical development: favipiravir, an inhibitor of a viral RNA-dependent RNA polymerase showing activity against many RNA viruses, as well as ritonavir and lopinavir, both HIV-1 protease inhibitors [8,9]. However, the so far largest randomized evaluation of COVID-19 therapy (RECOVERY) study revealed the only therapeutic benefit for low dose treatment with dexamethasone, while all other repurposed drugs failed [10]. This included hydroxychloroquine (H-CQN) and chloroquine (CQN). Both did also show no benefit in hospitalized patients with COVID-19 [10].

**Figure 1:**
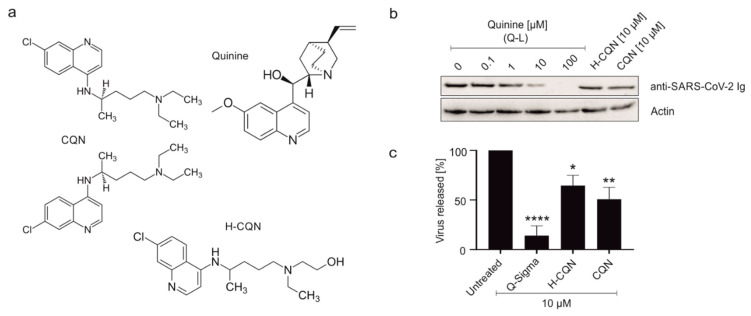
Influence of quinine, H-CQN, and CQN on the replication of SARS-CoV-2 in Vero B4 cells. (**a**) Molecular structures of quinine, H-CQN, and CQN. (**b**) Western blot analysis of virus fractions (anti-SARS-CoV-2 Ig). The cell fractions were stained with ß-actin antibody. Quinine-sulfate was used as (**b**) Q-L or (**c**) Q-S. Cell culture supernatants were harvested at 3 d post-infection (dpi). The virions were purified and analyzed by Western blot using a SARS-CoV-2 convalescent serum. (**c**) Densitometric analysis of Western blot analysis was performed using AIDA^®^. Analysis of five independent experiments ±standard deviation (SD) (Q-S **** *p* < 0.001 using a one-sample *t*-test), three independent experiments ±SD (H-CQN * *p* = 0.0270), or four independent experiments ±SD (CQN ** *p* = 0.0039).

After first promising in vivo and in vitro results, the lack of antiviral activity of H-CQN has been reported for hospitalized patients with COVID-19 by multiple double-blinded clinical trials [5,11,12,13]. Similarly, H-CQN was ineffective in non-human primates [14] and several animal models [15]. Limited antiviral activity of H-CQN and CQN was shown in Calu-3 [16,17] and human airway epithelium cells [14]. Very recently, it was shown that the antiviral effect of H-CQN can be augmented by combinational treatment with TMPRSS2 inhibitors [16]. The non-hydroxy analog CQN was also ineffective in preventing SARS-CoV-2 infection in human lung cells depending on the expression of the protease TMPRSS2 [17]. CQN and H-CQN (Figure 1a) are commonly used for malaria treatment and for autoimmune diseases, e.g., rheumatoid arthritis and systemic lupus erythematosus [18,19]. Like other antimalarial drugs, they have also been evaluated as antivirals for various viral diseases [20].

Mechanistically, H-CQN and CQN were shown to increase the pH of acidic organelles, such as endosomes, thereby interfering with the receptor-mediated endocytosis of viruses [21,22]. They were also shown to act on lymphocytes and macrophages, leading to a reduction in an inflammatory response, which might suppress the cytokine storm associated with the COVID-19 progression [21]. However, the potential of a therapeutic effect of H-CQN and CQN remains controversially discussed in the light of severe side effects associated with these drugs, e.g., gastrointestinal problems, visual and hemogram disorders, cramps, cardiac arrhythmias (prolonged QT syndrome), and retinopathy [23,24,25].

Given the fact that H-CQN and CQN originated from the natural substance quinine, we hypothesized that quinine might exert antiviral activity against SARS-CoV-2. We hence tested quinine in two versions: (i) quinine-sulfate extracted from a tablet (Limptar N, Cassella Med GmbH), termed Q-Limptar (Q-L), and (ii) quinine-sulfate as solid material directly obtained from a supplier (Sigma), termed Q-Sigma(Q-S). Our data suggest that quinine exerts antiviral activity against SARS-CoV-2 with lower cytotoxicity as compared to H-CQN and CQN and that this antiviral effect occurs in several TMPRSS2+ human cancer cell lines.

## 2. Materials and Methods

### 2.1. Viruses

From a 61-year-old patient, nasal and pharyngeal swab samples were taken 6 days after the presumed date of infection and 2 days after the start of mild symptoms of COVID-19. This first swab was positive by real-time-PCR with Ct value of 15, further follow-up swab after 10 days was still positive with Ct value of 31, and further follow-up swabs 18, 19, and 35 days after the first swab remained negative [26].

The virus strain SARS-CoV-2_PR1_ was amplified in Vero B4 cells. Viral titers were determined by an endpoint titration assay. For the generation of new virus stock, virus-containing cell culture supernatant was harvested at 72 h post-infection (hpi) and passed through a 0.45 μm pore-size filter. Virus stocks were stored at −80 °C until further usage. For Western blot analysis, Vero B4 cells were infected with SARS-CoV-2_PR1_ (multiplicity of infection (MOI) = 0.008) for 1 h and further treated with interventions. At 72 hpi, virus-containing cell culture supernatants were harvested, and released virions were purified through 20% (*w*/*v*) sucrose cushion (20,000× *g*, 4 °C, 90 min). For MOI determination of SARS-CoV-2_PR1_ virus stocks, Vero B4 cells were infected with serial dilutions of the virus stock over 72 h. Afterward, cells were fixed (4% PFA), permeabilized (0.5% Triton/PBS), blocked (1% BSA/PBS-T), and finally stained with a SARS-CoV-2 NP antibody (Biozol, Eching, Germany). The endpoint of virus infection was analyzed via fluorescence microscopy, and viral titer was calculated by the method of Reed and Muench [27].

The recombinant SARS-CoV-2 expressing mNeonGreen (icSARS-CoV-2-mNG) [28] was obtained from the World Reference Center for Emerging Viruses and Arboviruses (WRCEVA) at the University of Texas Medical Branch (UTMB). To generate icSARS-CoV-2-mNG stocks, Caco-2 cells were infected, the supernatant was harvested at 48 hpi, centrifuged, and stored at −80 °C. For MOI determination, a titration using serial dilutions of the virus stock was conducted. The number of infectious virus particles per mL was calculated as the (MOI × cell number)/(infection volume), where MOI = −ln(1 − infection rate).

### 2.2. Cell Culture

Vero B4 cells were maintained in Dulbecco’s Modified Eagle’s Medium (DMEM) containing 10% (*v*/*v*) inactivated fetal calf serum (FCS), 2 mM l-glutamine, 100 U/mL penicillin, and 100 μg/mL streptomycin.

Caco-2 (human colorectal adenocarcinoma) cells were cultured at 37 °C with 5% CO_2_ in DMEM containing 10% FCS, with 2 mM l-glutamine, 100 µg/mL penicillin-streptomycin, and 1% NEAA. A549 cells were cultured at 37 °C with 5% CO_2_ in RPMI-1640 containing 10% FCS, with 100 µg/mL penicillin-streptomycin.

Calu-3 (human lung adenocarcinoma) cells were cultured at 37 °C with 5% CO_2_ in DMEM containing 10% FCS, with 2 mM l-glutamine and 100 µg/mL penicillin-streptomycin.

A549-cells expressing ACE2 and TMPRSS2 were generated by retroviral transduction. Briefly, 293T-cells were seeded in 6-well plates and transfected with a lentiviral construct expressing hACE2 and puromycin with an IRES (pLV-EF1a_hACE2_IRES-puro, kindly provided by Daniel Sauter, Ulm) and helper plasmids pVSV-G and psPAX2 by using polyethylenimmine (PEI). In addition, we generated MLV-based retroviral particles expressing TMPRSS2 by using pQCXIBL_hTMPRSS2_c-myc-BlaR and helper plasmids pVSV-G and pMLV-gag/pol. After 24 h, the medium containing the virus was collected and centrifuged at 3200× *g* for 10 min. The supernatant was transferred to a new collection tube and stored at 4 °C. A549 seeded in a T25 flask were then transduced with 2 mL virus-containing supernatant and cultured for 14 days with 1.5 µg/mL puromycin in RPMI as a selection marker. After this period, ACE2-expression was confirmed by Western blot (data not shown). Then, ACE2-expressing A549 cells were further transduced with the MLV-based hTMPRSS2-expressing VLPs. The procedure was similar; however, cells were further treated with 5 µg/mL blasticidin as a resistance marker. Furthermore, using the same protocol, we generated A549-cells that were transduced with MLV-based pQCXIP_hACE2_Puro and pQCXIBL_hTMPRSS2_c-myc-BlaR from Markus Hoffmann (DPZ Göttingen, Germany). Altogether, we ended up with four A549 cell lines. They were termed A549-ACE2/TMPRSS2_c1 generated with the lentiviral ACE2 construct, A549-ACE2/TMPRSS2_c2 generated with an MLV-based construct and the two A549-ACE2 only expressing cell lines. For unknown reasons, only the A549-ACE2 generated with the lentiviral construct were permissive for infection in the absence of TMPRSS2, which is why we only used this cell clone for further experiments.

### 2.3. Determination of the Number of Viral RNA Copies from Released Viruses by qRT-PCR

The virus was quantified by real-time PCR AgPath-ID One-Step RT-PCR Kit from Ambion (Cat: 4387424), allowing reverse transcription, cDNA synthesis, and PCR amplification in a single step. Samples were analyzed by 7500 software v2.3 (Applied Bioscience). PCR primers were used according to [26]: RdRp_fwd: 5′-GTG-ARA-TGG-TCA-TGT-GTG-GCG-G-3′ and RdRp_rev 5′-CAR-ATG-TTA-AAS-ACA-CTA-TTA-GCA-TA-C-3′. The probe was 5′-CAG-GTG-GAA-/ZEN/CCT-CAT-CAG-GAG-ATG-C-3′ (label: FAM/IBFQ Iowa Black FQ). As a positive control, a specific target for the E and RdRp gene of SARS-CoV2 was used and made by Integrated DNA Technologies. Control: 5′-TAA-TAC-GAC-TCA-CTA-TAG-GGT-ATT-GAG-TGA-AAT-GGT-CAT-GTG-TGG-CGG-TTC-ACT-ATA-TGT-TAA-ACC-AGG-TGG-AAC-CTC-ATC-AGG-AGA-TGC-CAC-AAC-TGC-TTA-TGC-TAA-TAG-TGT-TTT-TAA-CAT-TTG-GAA-GAG-ACA-GGT-ACG-TTA-ATA-GTT-AAT-AGC-GTA-CTT-CTT-TTT-CTT-GCT-TTC-GTG-GTA-TTC-TTG-CTA-GTT-ACA-CTA-GCC-ATC-CTT-ACT-GCG-CTT-CGA-TTG-TGT-GCG-TAC-TGC-TGC-AAT-ATT-GTT-3′.

### 2.4. Inhibitors

Q-S as well as CQN were obtained from Sigma-Aldrich (St. Louis, MO, USA) and dissolved in DMSO. 200 mg Q-L (brand name: Limptar N, Cassella Med GmbH, Köln, Germany), was extracted from a tablet, pulverized, and dissolved in DMSO, resulting in a stock solution of 12.5 µM. H-CQN was acquired as a pure substance (Cayman, Ann Arbor, MI, USA) and dissolved in PBS, resulting in a stock solution of 11.5 mM. All interventions were used at the concentrations indicated in the different experiments.

### 2.5. Infection Experiments

Confluent monolayers of Vero B4, A549-ACE2, and A549-ACE2/TMPRSS2_c1 cells were infected in FCS-free DMEM with an MOI of 0.008 of the field isolate SARS-CoV-2PR-1 for 1 h. At 1 h post-infection (hpi), the inoculum was removed, and cells were treated with interventions. At 72 h post-infection, cells and supernatants were harvested. Cells were lysed in radioimmunoprecipitation assay (RIPA) buffer (150 mM NaCl, 50 mM Tris-HCl pH 8.0, 1% NP-40, 0.5% Na-deoxycholate, 0.1% sodium dodecyl sulfate (SDS), 10 mM ethylenediaminetetraacetic acid (EDTA)) containing protease inhibitor cocktail Complete (Roche, Basel, Switzerland), 5 mM N-ethylmaleimide (NEM), and 1 mM phenylmethylsulfonylfluoride (PMSF) and further used for Western blot analysis. Viral supernatants were either centrifuged by 20% sucrose cushion and analyzed via Western blot or incubated for 10 min at 95 °C and finally used for qRT-PCR analysis.

A total of 1 × 10^4^ Caco-2 or A549 cells/well were seeded in 96-well plates the day before infection in media containing 5% FCS. For Calu-3 cells, 4 × 10^4^ cells/well were used. Caco-2 cells were infected with the SARS-CoV-2 strain icSARS-CoV-2-mNG for 1 h at 37 °C at a multiplicity of infection (MOI) = 3 or mock-infected, A549 cells with MOI of 0.2 (low) or 1.1 (high), and Calu-3 cells with MOI of 0.8. At 1 hpi, the inoculum was removed, and the cells were treated with the inhibitors in concentrations from 0–100 µM during 48 h at 37 °C. A time-course of the infection was recorded using Cytation3 (Biotek, Winooski, VT, USA). Images of bright field and mNeonGreen (500,542) were taken every hour for 48 h at 4-fold magnification. After treatment, the cells were fixed with 2% PFA and stained with Hoechst (1 µg/mL final concentration). For quantification of infection rates, images were taken with Cytation3 (Biotek, Winooski, VT, USA), and Hoechst+ and mNeonGreen+ cells were automatically counted by the Gen5 Software (Biotek, Winooski, VT, USA).

### 2.6. SDS-PAGE and Western Blotting

Protein samples generated by infection experiments were separated by SDS-PAGE, transferred onto nitrocellulose membranes, blocked with 3% bovine serum albumin, and incubated with the appropriate primary antibody (Ab). Viral proteins were detected by antibodies derived from convalescent SARS-CoV-2 patient sera. The anti-β-actin antibody was purchased from Sigma-Aldrich (St. Louis, MO, USA). The anti-human and anti-rabbit secondary antibodies coupled to horseradish peroxidase (HRP) were obtained from Dianova (Hamburg, Germany).

For ACE2 detection, the proteins were separated by SDS-PAGE and transferred to PVDF-membrane. Blocking was performed with 5% milk. The anti-ACE2 antibody was purchased from AdipoGen life sciences (Liestal, Switzerland) and the anti-actin antibody from Sigma-Aldrich (St. Louis, MO, USA). The anti-mouse secondary antibody coupled to IRDye^®^-680RD was purchased from LI-COR (Lincoln, NE, USA).

### 2.7. Assessment of Cell Viability

The viability of uninfected cells was assessed by the water-soluble tetrazolium salt (WST)-1 assay (Roche) according to the manufacturer’s instructions. Cells were treated for 72 h with various inhibitors according to the protocols of the infection experiments.

### 2.8. Quantitative Real-Time PCR

RNA was extracted from A549 cell lines using the Qiagen RNeasy Mini Kit. Reverse transcription was performed with the Qiagen QuantiTect Reverse Transcription Kit using primers indicated in Table 1. For the qPCR, the New England Biolabs Luna Universal qPCR Master Mix was used. The reactions were performed following the manufacturers’ protocols. The qPCR was conducted using the Roche LightCycler 480 II.

### 2.9. Software and Statistics

We used Microsoft Word and Excel. GraphPad Prism 8.0 was used for statistical analyses and to generate graphs. Figures were generated with CorelDrawX7. Other software used included Gen5 v.3.04.

## 3. Results

### 3.1. Quinine Inhibits SARS-CoV-2 More Potently as Compared to H-CQN and CQN in Vero B4 Cells

In order to analyze if quinine displays antiviral activity against SARS-CoV-2, first, Vero B4 cells were infected with SARS-CoV-2_PR1_ and treated with quinine, either as Q-L or as Q-S, and, as a control, with H-CQN or CQN (Figure 1a). Three days post-infection (dpi), cell culture supernatants were harvested, and virus production was analyzed by Western blot (Figure 1b). Treatment with quinine leads to a strong reduction of SARS-CoV-2 replication. At 10 µM, the production of progeny virions was almost completely blocked (Figure 1b,c). The level of antiviral activity was independent of whether quinine was added as Q-L (Figure 1b) or Q-S (Figure 1c). Of note, quinine displays an even higher efficacy in the inhibition of SARS-CoV-2 replication than H-CQN or CQN: at 10 µM quinine, the replication was reduced by up to 90%, whereas 10 µM H-CQN led to a reduction of ~50% (Figure 1c). Thus, these data implicate that quinine exhibits antiviral activity against SARS-CoV-2, which is in Vero B4 cells even more potent than the described antiviral activities of H-CQN and CQN [17].

**Figure 2:**
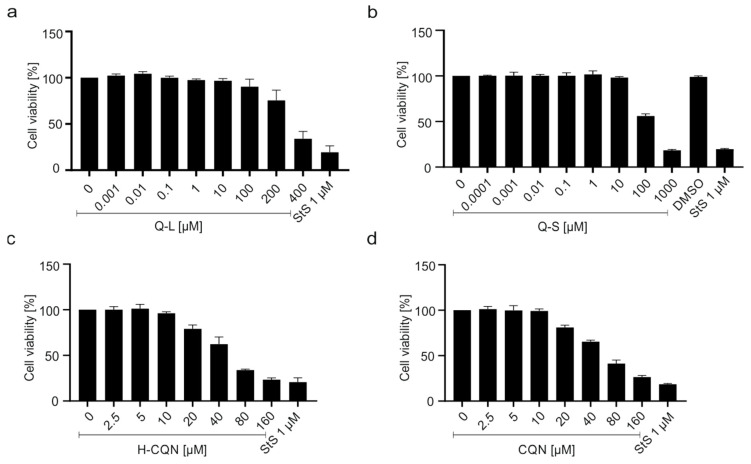
Influence of quinine (**a**,**b**), H-CQN (**c**), and CQN (**d**) on the cell viability of Vero B4 cells. Cells were treated for 72 h with (**a**) Q-L, (**b**) Q-S, (**c**) H-CQN, and (**d**) CQN to the protocols of the infection experiments. The influence on cell viability was measured by water-soluble tetrazolium salt (WST)-1 assays. Quinine-sulfate was used as (**a**) Q-L or (**b**) Q-S. Bars represent mean values of 3 independent experiments ±SD.

To control for potential unspecific effects of drug treatment on cell viability, water-soluble tetrazolium salt (WST)-1 assays were performed in uninfected cells treated with increasing concentrations of the drugs. Treatment at concentrations that effectively suppressed SARS-CoV-2 replication had no impact on cell viability (Figure 2a,b). In Vero B4 cells, the TD_50_ was ~300 µM for Q-L (Figure 2a) and ~100 µM for Q-S (Figure 2b), which is again better than that of H-CQN and CQN (~50 µM) (Figure 2c,d).

### 3.2. Quinine Inhibits SARS-CoV-2 Infection and Spread in Human TMPRSS2^+^ Colon Cells

**Figure 3:**
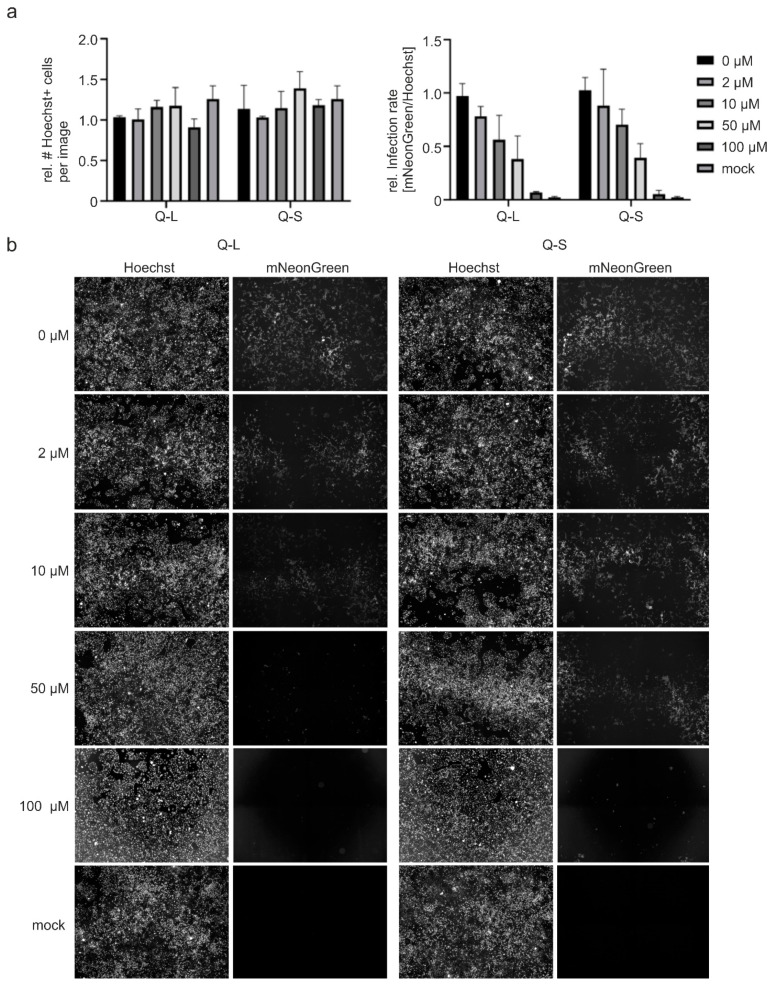
Influence of quinine on SARS-CoV-2-mNG infection in Caco-2 cells. Cells were infected with SARS-CoV-2-mNG at an MOI of 3 for 1 h or mock-infected. Then the inoculum was removed, and the cells were treated with the indicated amounts of Q-L or Q-S. At 48 h post-infection (hpi), cells were fixed with 2% PFA and nuclei were stained with 1 µg/mL Hoechst. The total amount of cells (Hoechst+) and infected cells (mNeonGreen+) were analyzed by automated microscopy. (**a**) Quantitative analyses of total cells and infected cells. Mean values and SD are calculated from two experiments with duplicate infections. (**b**) Representative fluorescence microscopy images taken at 4-fold magnification.

**Figure 4:**
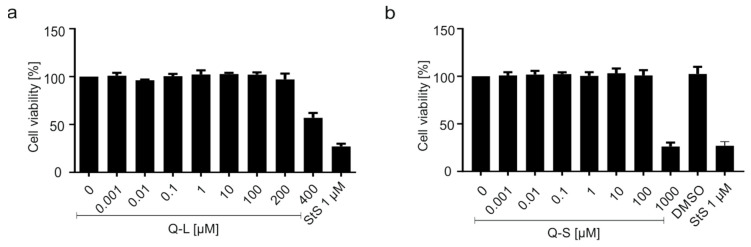
Influence of quinine (a + b) on the cell viability of CaCo-2 cells. Similar to the drug treatment scheme described in Figure 2, the influence on cell viability was measured by WST-1 assays. Quinine-sulfate was used as (**a**) Q-L or (**b**) Q-S. Bars represent mean values of 3 independent experiments ±SD.

We next aimed to validate the finding that quinine has antiviral activity in a SARS-CoV-2 permissive human cell system. For this, we employed human Caco-2 colon carcinoma-derived epithelial cells expressing TMPRSS2, a protease required for virus entry [29,30] and that was reported to restrict responsiveness to SARS-CoV-2 infection toward treatment with CQN [16,17]. To facilitate the detection and analyses of infected cells, as well as to quantify viral spread in living cells, we used the recombinant SARS-CoV-2 infectious clone, icSARS-CoV-2-mNG, expressing mNeonGreen as a reporter gene [28]. For a robust fluorescence readout, we infected Caco-2 cells in 96-well plates with a high MOI of 3. Afterward, cells were treated with different amounts of Q-L and Q-S. At 48 hpi, cells were fixed and nuclei were stained with Hoechst in order to determine the relative infection rate (mNeonGreen+/Hoechst+ cells) as well as any potential toxic effects of the treatment and infection (Figure 3). As for Vero B4 cells (Figure 2), the effect of the compounds on cell viability was further analyzed by WST-assays at a dose range up to 100 µM (Figure 4). Both Hoechst-staining as well as WST-assays confirmed our results obtained from Vero cells suggesting that concentrations up to 100 µM of Q-L and Q-S are non-toxic for Caco-2 cells (Figure 3a and Figure 4). Furthermore, Q-L or Q-S treatment in doses of 50 µM and above inhibited SARS-CoV-2 infection at this high MOI setting nearly completely, with a dose-dependent effect down to 2 µM (Figure 3).

**Figure 5:**
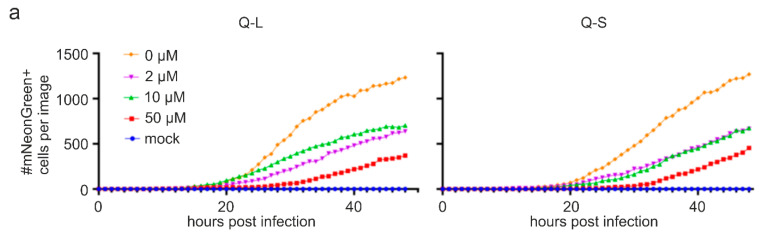

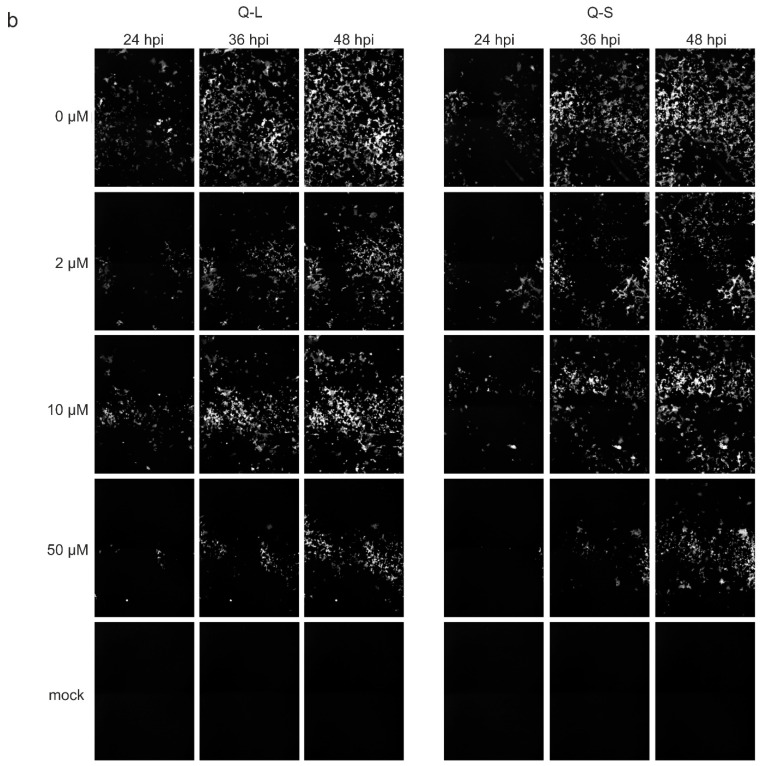
Influence of quinine on the replication of SARS-CoV-2-mNG in Caco-2 cells. Similar experimental layout as described in the legend of Figure 3. However, cells were imaged every hour post removal of the virus, and the number of infected cells (mNeonGreen+) was analyzed by automated microscopy. (**a**) Quantitative analyses of the time-course of viral replication as determined by the amount of mNeonGreen+ cells over 48 h. (**b**) Representative fluorescence microscopy images for 24, 36 and 48 hpi of mNeonGreen+, i.e., SARS-CoV-2-mNG-infected cells taken at 4-fold magnification. The full-length time-course analyses are available as Supplemental Videos (S1 and S2).

Taking advantage of the possibility to monitor viral spread in living cells, we imaged cells infected with icSARS-CoV-2-mNG and treated with Q-L/Q-S over a period of 48 h in 1 h intervals (Figure 5). Of note, quinine treatment strongly delayed as well as suppressed viral spread and SARS-CoV-2 replication in Caco-2 cells (Figure 5 as well as Supplemental Movies S1 and S2). Hence, quinine is effective against SARS-CoV-2 in human colon cells, and this inhibitory effect occurs early during infection and even at high virus doses of initial infection.

### 3.3. Quinine Restricts Viral Infection in Human Transgenic Lung Cancer Cells

Furthermore, we aimed to confirm our findings in human lung alveolar basal epithelial cells, as lung cells are the major host cells for SARS-CoV-2 infection in vivo. For this, we employed A549 cells, which were generated from a human lung adenocarcinoma [31]. As these cells usually do not express the ACE2 receptor and the TMPRSS2 protease necessary for SARS-CoV-2 entry, we generated cell lines stably expressing either the receptor ACE2 or both ACE2 as well as TMPRSS2. We used two different ACE2 constructs to generate stable cell lines, which were either termed A549-ACE2/TMPRSS2_c1, using a lentiviral ACE2 construct, or A549-ACE2/TMPRSS2_c2, using an MLV-based construct. Expression of ACE2 and TMPRSS2 in the transgenic cell lines was confirmed by Western blot (Supplementary Figure S1a) and qRT-PCR (Supplementary Figure S1b). Notably, only A549-ACE2 generated with the lentiviral construct were permissive for infection in the absence of TMPRSS2, which is why we only employed one cell clone that expresses ACE2 alone in subsequent experiments.

**Figure 6:**
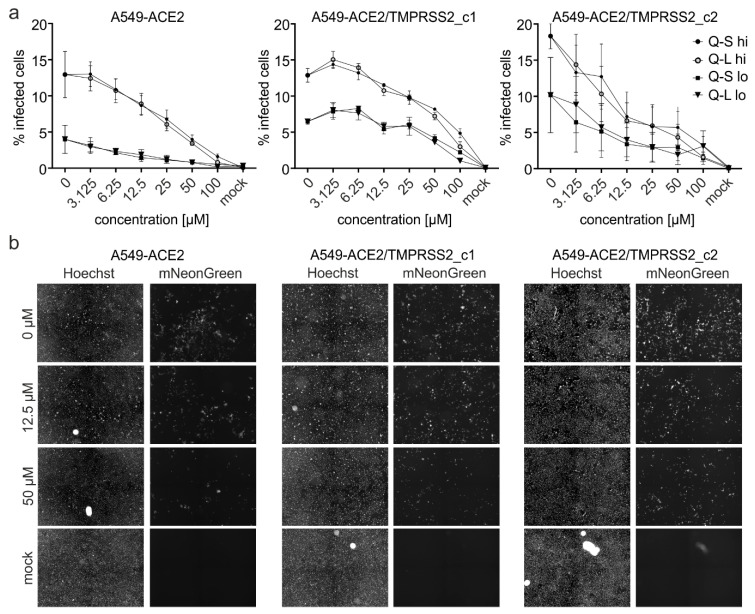
Influence of Q-S, Q-L on SARS-CoV-2-mNG infection in ACE2 and TMPRSS2-expressing A549 cells. A549-stably transduced to express ACE2, ACE2/TMPRSS2 clone 1, and ACE2/TMPRSS2 clone 2 were infected with SARS-CoV-2-mNG at high and low MOIs (see M&M) for 1 h or mock-infected. Then the inoculum was removed, and the cells were treated with the indicated amounts of Q-S and Q-L. At 48 hpi, cells were fixed with 2% PFA and nuclei were stained with 1 µg/mL Hoechst. (**a**) The total amount of cells (Hoechst+) and infected cells (mNeonGreen+) were analyzed by automated microscopy to calculate the absolute % of infected cells. Mean values and SEM are calculated from three independent experiments. (**b**) Representative fluorescence microscopy images (4-fold magnification) of high titer infection of the respective cell line treated with the indicated amounts of Q-L.

As for Caco-2 cells (Figure 3 and Figure 5), icSARS-CoV-2-mNG expressing mNeonGreen as a reporter gene was used to quantify viral infection. A549-ACE2, A549-ACE2/TMPRSS2_c1, and c2 were infected with different doses of icSARS-CoV-2-mNG and treated with different amounts of Q-L and Q-S. At 48 hpi, cells were fixed and nuclei were stained with Hoechst in order to determine the relative infection rate (mNeonGreen+/Hoechst+ cells) (Figure 6).

Treatment with Q-L or Q-S in doses of 50 µM and above inhibited SARS-CoV-2 infection nearly completely, with a dose-dependent effect down to 3.125 µM (Figure 6). Importantly, while the expression of TMPRSS2 lowered the antiviral activity of quinine in one cell clone, it did not cause complete loss of activity, as it was demonstrated by others for CQN [17]. Even though, and as expected, A549-cells expressing TMPRSS2 in addition to ACE2 allowed for higher infection rates, the antiviral activity of quinine was clearly detectable with a nearly complete block of infection at high concentrations. The IC_50_ varied strongly dependent on the cell clone and the MOI and was in the range of ~7.5 to ~50 µM for high and ~3.7 to 50 µM for low virus titers (compare Figure 6a and Table 2).

**Figure 7:**
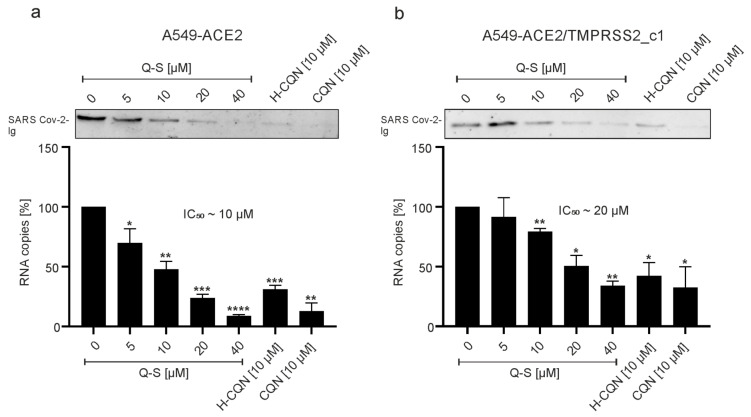
Influence of quinine, H-CQN, and CQN on the replication of SARS-CoV-2 in ACE2- and TMPRSS2-expressing A549 cells. Western blot analyses and q-RT-PCR of virus fractions in (**a**) ACE2- and (**b**) ACE2/TMPRSS2_c1-expressing A549 cells with corresponding IC_50_ values of Q-S. For both cell lines, cell culture supernatants were harvested at 3 dpi. The virions were either purified and analyzed by Western blot using a SARS-CoV-2 convalescent serum (upper inserts) or analyzed by q-RT-PCR. PCR results are shown as analysis of three independent experiments ±standard deviation (SD). * *p* ≤ 0.05, ** *p* < 0.005, *** *p* < 0.0009 and **** *p* < 0.0001 using a one-sample *t*-test.

To further evaluate the antiviral effect of the compounds, A549-ACE2 or A549-ACE2/TMPRSS2_c1 were infected with SARS-CoV-2_PR1_ and treated with different concentrations of Q-S, H-CQN, or CQN. At 3 dpi, cell culture supernatants were harvested, and virus production was analyzed by Western blot and qRT-PCR (Figure 7). Consistent with the results obtained with SARS-CoV-2 mNG, treatment with quinine leads to a strong reduction of SARS-CoV-2 replication in both ACE2 and ACE2/TMPRSS2-expressing A549 cells. In A549-ACE2 cells, 40 µM of Q-S led to a nearly complete restriction of progeny virions, while the same treatment of cells expressing ACE2 and TMPRSS2 led to a reduction of only 40% (Figure 7). Of note, H-CQN and CQN were more effective in this system than Q-S: at 10 µM quinine, the replication was reduced by 50% in ACE2 positive cells and 25% in TMPRSS2 expressing cells, whereas 10 µM CQN led to a reduction of either 80% or 65% (Figure 7).

**Figure 8:**
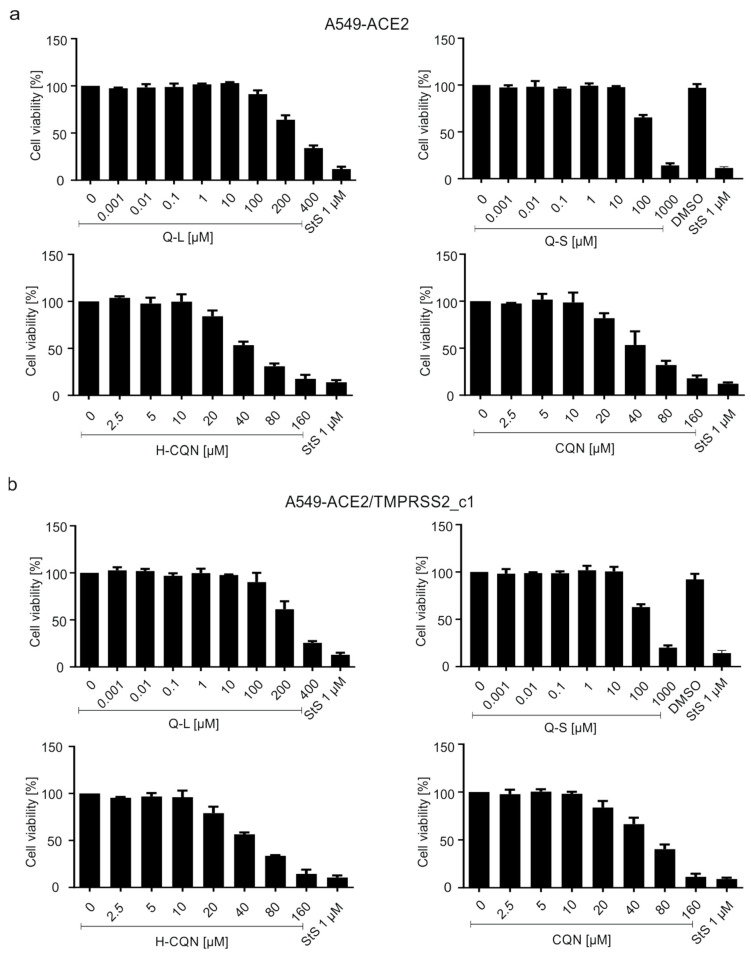
Influence of quinine, H-CQN, and CQN on the cell viability of A549-ACE2 and A549-ACE2/TMPRSS2 cells. Similar to the drug treatment scheme described in Figure 2, the influence on cell viability was measured in (**a**) A549-ACE2 or (**b**) A549-ACE2/TMPRSS2_c1 cells by WST-1 assays. Quinine-sulfate was used as Q-L or Q-S. Bars represent mean values of 3 independent experiments ±SD.

To exclude unspecific cytotoxic effects of the compounds, WST-1 assays were conducted (Figure 8). Thereby, concentrations used for antiviral treatment had no effect on cell viability in A549-ACE2 (Figure 8a) or A549-ACE2/TMPRSS2_c1 (Figure 8b). The TD_50_ values were ~300 µM for Q-L and ~150 µM for Q-S, which is better than that of H-CQN and CQN (~40–50 µM) (Figure 8).

Conclusively, the results implicate that quinine exhibits antiviral activity against SARS-CoV-2 in A549 lung cancer cell lines and that its antiviral activity might be modulated but not abrogated by the expression of TMPRSS2.

### 3.4. Quinine Inhibits SARS-CoV-2 Infection in Human Calu-3 Lung Cells

**Figure 9:**
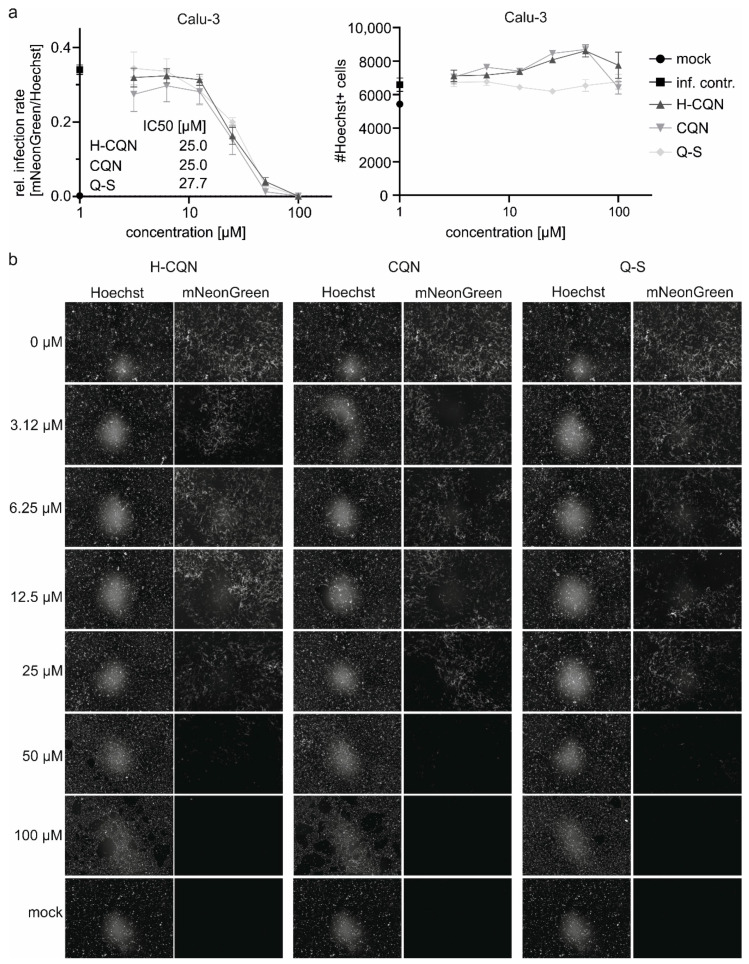
Antiviral activity of H-CQN, CQN, and quinine on SARS-CoV-2-mNG infection in Calu-3 cells. Cells were infected with SARS-CoV-2-mNG at an MOI of 0.2 for 1 h or mock-infected. Then the inoculum was removed, and the cells were treated with the indicated amounts of H-CQN, CQN, or Q-S. At 48 hpi, cells were fixed with 2% PFA and nuclei were stained with 1 μg/mL Hoechst. The total amount of cells (Hoechst+) and infected cells (mNeonGreen+) were analyzed by automated microscopy. (**a**) Quantitative analyses of infection rates and the total number of cells for the treatment with the different compounds. Mean values and SEM are calculated from three independent experiments with triplicate infections. (**b**) Representative fluorescence microscopy images taken at 4-fold magnification.

The best characterized and most extensively studied surrogate lung cell infection model for SARS-CoV-2 are Calu-3 cells expressing ACE2 and TMPRSS2 endogenously [32]. We infected Calu-3 with icSARS-CoV-2 mNG and treated the cells with different concentrations of H-CQN, CQN, and Q-S (Figure 9). Consistent with previous cell systems (Figure 1, Figure 3, Figure 6, and Figure 7) also in this model, quinine exerted antiviral activity (IC50 27 µM), which was comparable to that of H-CQN and CQN (IC50 for both 25 µM).

Altogether, even though we detected differences in the IC50 values dependent on the cell line and the MOI used, our cumulated data demonstrate that in various cancer cell lines, H-CQN, CQN as well as quinine exert antiviral activity against SARS-CoV-2 and that those antiviral activities occur in the presence of TMPRSS2.

## 4. Discussion

The natural substance quinine exerts antiviral activity against SARS-CoV-2 that was comparable to its chemical derivatives, H-CQN and CQN, particularly in TMPRSS2+ human lung cancer cell lines. This is of importance, as it was suggested that TMPRSS2 expression might be one factor limiting the effectivity of CQN [17]. However, we found that the antiviral activity of this class of compounds is not abrogated by the expression of TMPRSS2.

Originally, H-CQN was first shown to interfere with SARS-CoV-2 replication in vitro [33], and soon in the pandemic, H-CQN and CQN have been tested most intensively in more than 80 clinical studies leading to different regional recommendations for treatment of hospitalized COVID-19 patients [11,13,33,34,35,36,37,38,39,40,41,42]. In the U.S., H-CQN and CQN were fast-tracked through clinical trials in order to determine their efficacy in the treatment of COVID-19, although this recommendation has been retracted nowadays [43].

Quinine is being tested in clinical studies for its antiviral effect against SARS-CoV-2: In Indonesia, health authorities are trialing quinine as a possible treatment of COVID-19 [44]. In Russia, the Federal Biomedical Agency wants to test the quinine derivative mefloquine in clinical trials [45]. In the US, a randomized, placebo-controlled study assesses the effect of a quinine-containing nasal spray in healthcare professionals [46]. Although there is first enigmatic in vitro data that quinine and analogs restrict SARS-CoV-2, neither clinical nor preclinical evidence for any potential therapeutic activity of quinine against this virus has been reported so far [47].

Looking at the history, H-CQN and CQN are derivatives from their natural predecessor quinine, an extract of the bark of the Chinchona tree (native to the Andes of South America), that was used to treat feverish infections, particularly malaria, for hundreds of years almost worldwide [43,48]. Quinine and quinidine, both content of the Chinchona bark, are alkaloids and stereoisomers of each other [43]. The first records for medical use of quinine date back to 1630, where in Peru, the countess of Chinchon developed malaria and was successfully treated with an extract of the bark of the fever tree, which was later termed Chinchona bark [49]. First isolated in 1820 and chemically synthesized in 1944, it was the only antimalarial drug available [49]. In the 19th century, British citizens and soldiers used tons of the Chinchona bark to protect themselves from malaria and thus permitted a stable British population in tropical colonies. Based on this natural product, H-CQN was synthesized in 1946 and mainly used for the treatment of malaria. Nevertheless, and even until now, quinine is used for the treatment of severe and H-CQN-resistant cases of malaria tropica [50].

It is also approved for the treatment of calf cramps, and it is commonly used as an aromatic agent. For instance, it is added to bitter lemon and tonic water. Quinine has been commercialized since the 19th century in beverages known as Indian quinine tonic. The latter was the standard beverage for the pioneers in malaria-invested tropical areas. Particularly British soldiers mixed tonic water with lime and gin. Winston Churchill said: “*The gin and tonic has saved more Englishmen’s lives, and minds, than all the doctors in the Empire*” [51].

Quinine can be added up to 100 mg/kg for food or up to 85 mg/L to beverages and up to 250 mg/L to alcoholic beverages [52]. Quinine has antimicrobial activities useful for the treatment of bacteria and viruses. For instance, it has been shown in vitro that quinine has antiviral activity against dengue, herpes simplex, and influenza A viruses [53,54,55].

Facing the recent SARS-CoV-2 and possible future pandemics, there is the general challenge to bridge the gap between the emergence of the virus and the development of specifically and directly acting antiviral drugs and antibodies. In the early face of a pandemic where only hygienic measures and confinements are the options to stop the spread of infections among the affected population, either repurposed synthetic drugs or natural substances with a broadly acting antiviral capacity are the only options for therapeutic intervention. Similar to quinine, the natural substance iota-carrageenan was shown to interfere with SARS-CoV-2 replication in vitro [56,57,58]. For this polysaccharide isolated from red algae, which can be used as a prophylactic nasal and throat spray or as a lozenge, first clinical trials in Austria, the United States, Argentina, and Great Britain have been reported, and first positive results have been published [56,59,60].

Medical analysis showed that high levels of cytokines in the plasma of patients correlate with a severe SARS-CoV-2 infection, assuming that an enhanced cytokine storm could be responsible for the need to transfer severe COVID-19 cases to the intensive care unit [1]. It was previously demonstrated that H-CQN and CQN have the ability to reduce the inflammatory response and thus have antipyretic activity [21]. In addition, quinine with a long-standing record as medication against feverish illnesses might be able to mitigate the cytokine storm associated with severe COVID-19.

In addition, as an approved aromatic agent, quinine is added to bitter lemon and tonic water. According to the well-elaborated pharmacokinetics, 85 mg quinine, present in 1 L of, e.g., tonic water, could lead to a plasma concentration of ~0.5 µg/mL, which corresponds to a molarity of ~1.5 µM [61,62], which would reach values that show already some efficacy in our in vitro systems.

Notably, H-CQN and quinine have different pharmacokinetics. According to in vivo studies, treatment results in an estimated 20 times higher plasma concentration for quinine than for H-CQN [63,64]. For instance, a 200 mg dose translates into plasma molarity of ~0.15 µM for H-CQN and ~2.9 µM for quinine [62,65]. Thus, quinine appears to exert a clear antiviral effect against SARS-CoV-2 with a significantly better toxicity profile in vitro and has a predictable better plasma availability when compared to H-CQN and CQN. Indeed, there are rudimentary reports about first cases of total recovery after treatment with quinine [66].

If SARS-CoV-2 caused a pathogenic profile similar to typical seasonal coronaviruses without transition into the severe COVID-19 stage, the world would be enfranchised from one of its most serious current problems. Thus, there is a tremendous medical need to prevent the infection and, if occurred, the transition of a mild SARS-CoV-2 infection into a severe COVID-19 stage by having access to a safe, relatively cheap, and easily distributable drug. Altogether, if our data would hold up in further clinical studies, it would be legitimate to conclude that the usage of quinine is an alternative option for the treatment of SARS-CoV-2 infections.

## Figures and Tables

**Table 1:** Primer sequences used for quantitative real-time PCR.

Primer Name	Sequence
qRT hGAPDH_fwd	tgc acc acc aac tgc tta gc
qRT hGAPDH_rev	ggc atg gac tgt ggt cat gag
qRT_hACE2 fwd	gat gcc tcc ctg ctc att tg
qRT_hACE2 rev	aac ttc tcg gcc tcc ttg aa
qRT_hTMPRSS2 fwd	agg acg aga atc ggt gtg tt
qRT_hTMPRSS2_ rev	gga tcc gct gtc atc cac ta

**Table 2:** IC_50_ (µM) values of Q-L and Q-S in A549-cells stably expressing ACE2 and TMPRSS2. The IC_50_ [µM] was calculated with GraphPad Prism (log(inhibitor) vs. response—variable slope (four parameters)). MOI for hi is 1.1 and lo is 0.2.

Compound IC_50_ [µM]	A549-ACE2		A549-ACE2/TMPRSS2_c1		A549-ACE2/hTMPRSS2_c2	
	hi	lo	hi	lo	hi	lo
Q-S	29.07	5.98	50.02	52.82	7.48	3.75
Q-L	24.42	13.35	55.82	52.86	7.50	5.58

## Data Availability

All study data are included in the article.

## Supplementary Materials

The following are available online at https://www.mdpi.com/article/10.3390/v13040647/s1, Supplementary Figure S1, Video S1: SMovie2_Q-L 0 and 50 uM, Video S2: SMovie1_Q-S 0 and 50 Um.
