# Supplementary material for: Quinine Inhibits Infection of Human Cell Lines with SARS-CoV-2
Source: Viruses. 2021 Apr 9;13(4):647. doi: 10.3390/v13040647 (PMC8069458; doi:10.3390/v13040647)
Supplement: Supplementary file 1 [file viruses-13-00647-s001.zip › Supplementary Figure 1.docx]

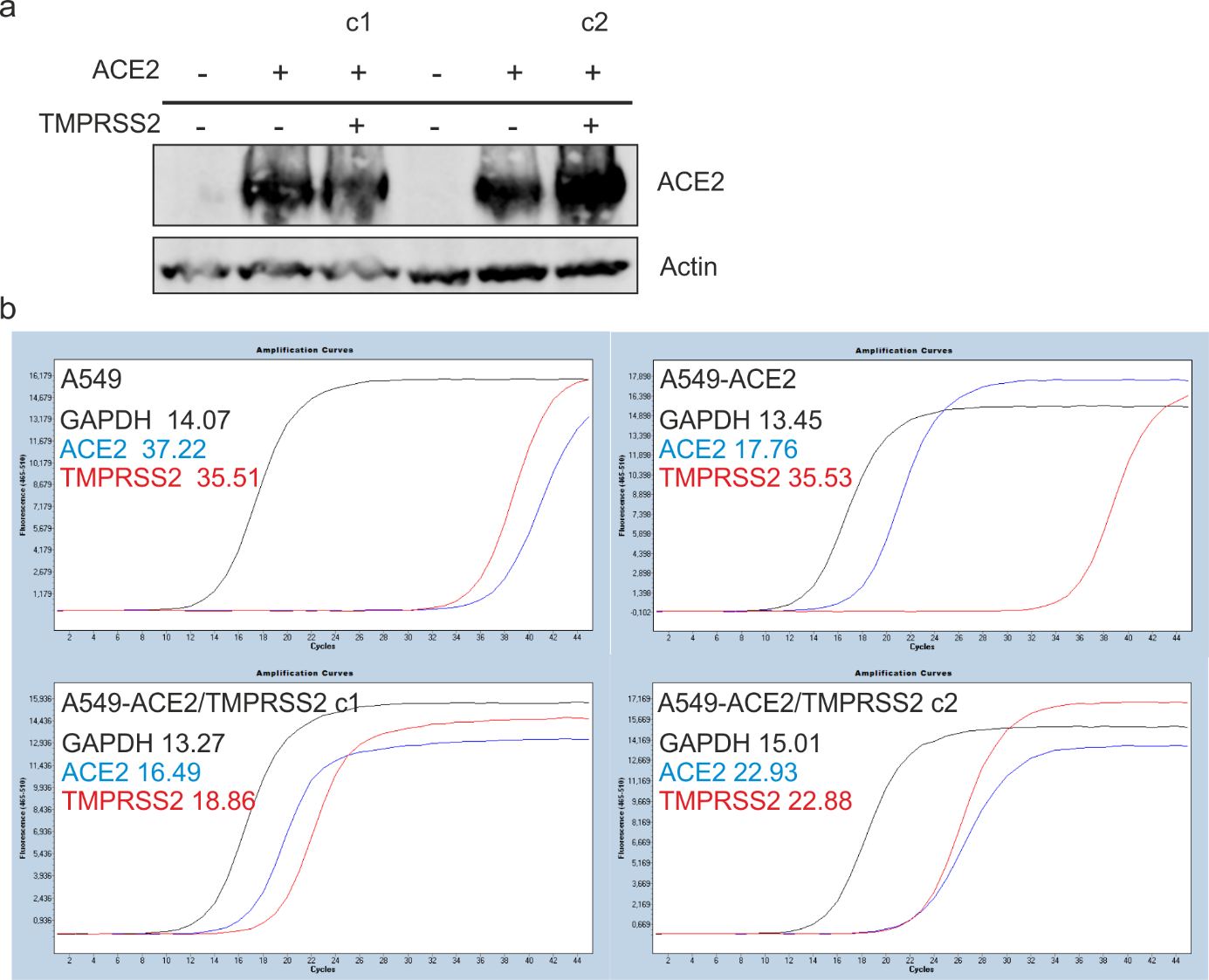


**Supplementary Figure 1. ACE2 and TMPRSS2 expression analysis in parental A549, ACE2 and ACE2/TMPRSS2 cells.** Cell lysates for Western blotting and RNA for qRT-PCR of the various cell lines was obtained and analyzed as detailed in the M&M section. (a) Steady-state expression of ACE2 in the various A549-transgenic cell lines and the parental clone and (b) primary melting curves and Cp-values of the qRT-PCR analyses of GAPDH as housekeeping gene (black), ACE2 (cyan) and TMPRSS2 (red). The Western blot analyses was done three independent times and the qRT-PCR shows one representative example out of two independent measurements.
